# Stress, Health, and Injury Among Illinois Farmers

**DOI:** 10.1002/ajim.70000

**Published:** 2025-07-02

**Authors:** Josie M. Rudolphi, Salah Issa, Courtney Cuthbertson, Kaleigh Barnett

**Affiliations:** ^1^ University of Illinois Urbana‐Champaign Champaign Illinois USA

**Keywords:** agriculture, injury prevention, mental health, occupational health, occupational safety, stress

## Abstract

**Objectives:**

The goal of this cross‐sectional study was to characterize stress, injury, and chronic health conditions among agricultural producers in Illinois. The objectives were to: (1) describe the prevalence and nature of work‐related injuries; (2) describe chronic health conditions, stress, and symptoms of mental health conditions; and (3) explore relationships between work‐related injuries and stress, mental health, and health conditions.

**Methods:**

A cross‐sectional survey was conducted using a modified Dillman approach. Agricultural producers received mailed questionnaires assessing demographics, farm characteristics, chronic health conditions, stress (using the PSS questionnaire), anxiety (GAD‐7), depression (PHQ‐9), and work‐related injuries. Descriptive statistics describe the population, farm characteristics, injuries, symptoms of mental health, and chronic health conditions. Chi‐squared tests describe associations between variables.

**Results:**

Agricultural injuries were reported by 8.01% of respondents, and were primarily minor or moderate. Nearly half (49.07%) reported moderate to high stress, and 10.83% had symptoms of moderate to severe anxiety. No significant associations were found between injury and stress, anxiety, or depression.

**Conclusions:**

These findings highlight the complex interplay between health, stress, and safety in agricultural workers. Longitudinal and qualitative approaches are needed to better understand how stress and chronic conditions may relate to agricultural injuries.

## 1 Introduction

Agriculture is a cornerstone of the United States' economy, fueling domestic prosperity and ensuring global food security [1]. Yet, its pivotal role belies the stark reality that it is also one of the most hazardous industries. Agricultural producers and workers encounter a spectrum of occupational hazards—biological, chemical, ergonomic, physical, and psychosocial—that stem directly from their diverse work environments and daily tasks [2, 3, 4]. Consequently, these hazards are linked to a wide range of adverse health and safety outcomes [5, 6].

At the forefront of these adverse health and safety outcomes is agricultural work‐related injury—a critical concern in an industry where even a single incident can have lasting repercussions.

Agricultural work‐related injuries can be categorized into several types, including acute injuries, chronic conditions, and fatalities. Their incidence has varied across time and surveillance system [7]. In 2023, the average nonfatal workplace injuries and illnesses incidence rate within the United States agriculture, forestry, fishing, and hunting sector was 4.2 cases per 100 full‐time equivalent (FTE) workers [8]. The incidence rate for fatal work injuries in 2023 was 19.5 fatalities per 100,000 agricultural workers [9].

Agricultural work‐related injuries sustained on the job not only pose immediate threats to physical health [10, 11, 12, 13], but also are associated with chronic stress, anxiety, and depression [14, 15, 16], thereby exacerbating the overall burden on workers' well‐being. Producers not only endure the physical risks common to farm work but also shoulder significant decision‐making and managerial responsibilities, which may amplify the adverse effects of injuries on their mental health.

Considering these challenges, it is critical to understand how work‐related injuries may intersect with broader health outcomes among producers. This study describes agricultural work injuries, quantifies symptoms of mental health conditions, describes the prevalence of chronic health conditions, and considers associations between work‐related injuries and stress, mental health, and health conditions among Illinois farmers.

## 2 Materials and Methods

### 2.1 Study Design

This cross‐sectional study aimed to assess the prevalence of work‐related injuries, chronic health conditions, perceived stress, and mental health burdens among agricultural producers in Illinois. Details of participant recruitment and survey distribution have been described previously [17] and are summarized below.

### 2.2 Ethical Considerations

This study was approved by the University of Illinois Institutional Review Board. Written informed consent was obtained from all participants.

### 2.3 Sample and Recruitment

A random sample of 1000 addresses of agricultural producers were requested from US Farm Data (previously Farm Market iD [FMiD]) which maintains a database of agricultural producers with approximately 95% coverage [18]. Only records designating an individual as the primary contact were included to ensure targeted recruitment. Data were collected in two phases: Round 1 (June to August 2020) and Round 2 (March to May 2021).

### 2.4 Survey Distribution

To maximize response rates, a modified Dillman method [19] was employed. The initial mailing included a questionnaire, a detailed cover letter explaining the study aims and confidentiality measures, a list of mental health and stress management resources, a $5 cash incentive, and a prepaid return envelope. Three weeks after the initial mailing, a reminder postcard was sent to nonrespondents, followed by a final mailing 3 weeks later to all remaining nonrespondents; this final mailing duplicated the original package but did not include the $5 cash incentive.

### 2.5 Survey Instrument

The survey instrument was developed to capture both the psychological and physical aspects of agricultural work and included validated scales along with items tailored for the agricultural context.

### 2.6 Measures

#### 2.6.1 Demographic and Work‐Related Characteristics

Participants responded to items about their demographic background and work‐related characteristics. The data collected included gender, age, education level, ethnicity, race, marital status, number of children, years farming, employment type, farm type, farm role, and primary commodity.

#### 2.6.2 Perceived Stress Scale (PSS)

Stress was measured using the PSS, a widely used psychological instrument for measuring the perception of stress [20]. The scale consists of 10 items that ask respondents to rate the frequency of their stress‐related thoughts and feelings over the past month (i.e., “how often have you felt nervous or stressed?”). Responses are captured on a 5‐point Likert scale ranging from 0 (*never*) to 4 (*very often*). The scores are summed to yield a total stress score that ranges from 0 to 40, where higher scores indicate greater perceived stress [21].

The PSS is designed to be sensitive to chronic, ongoing stress rather than to a response to recent specific events, making it particularly useful in studies where the focus is on chronic stress assessment [22]. The PSS has been utilized in numerous studies to explore the relationship between perceived stress and various psychological and physiological outcomes [23, 24]. Psychometric evaluations of the PSS have demonstrated good reliability and validity [20, 21, 25]. In the current sample, the PSS demonstrated excellent internal consistency, with a Cronbach's alpha of *α* = 0.87.

#### 2.6.3 Patient Health Questionnaire (PHQ‐9)

Depression was measured using the PHQ‐9. The PHQ‐9 is designed to screen for the presence and severity of depressive symptoms in individuals [26, 27].

The instrument consists of nine items that participants answer based on the frequency of depressive symptoms they have experienced over the past 2 weeks (i.e., “little interest or pleasure in doing things”). Responses are recorded on a 4‐point Likert scale ranging from 0 (*not at all*) to 3 (*nearly every day*). The total score can range from 0 to 27, with the following severity level cut‐offs: 0−4 = minimal/none, 5−9 = mild, 10−14 = moderate, 15−19 = moderately severe, 20−27 = severe [28]. To assess the prevalence of likely clinical depression, a cut‐off score of ≥ 7 was used to define “probable depression,” based on evidence indicating this threshold provides a strong balance of sensitivity and specificity when compared to structured psychiatric diagnoses, particularly in population‐based screening contexts [29]. This categorization is used to identify individuals at risk of clinically significant depressive symptoms, even in the absence of a formal diagnosis.

The PHQ‐9 scale has demonstrated excellent reliability and validity [26, 28] across a variety of settings and populations [30] including agricultural environments [31, 32]. It has shown good psychometric properties, such as sensitivity and specificity [33].

#### 2.6.4 Generalized Anxiety Disorder Scale (GAD‐7)

Anxiety was measured using the GAD‐7. The GAD‐7 is a widely recognized self‐report tool designed to screen for GAD and assess the severity of anxiety symptoms [34].

Participants responded to seven items measuring symptoms of anxiety (i.e., “not being able to stop or control worrying”) based on their frequency over the past 2 weeks. Responses were captured on a 4‐point Likert scale ranging from 0 (*not at all*) to 3 (*nearly every day*). The total score can range from 0 to 21, with the following severity cut‐offs: 0−4 = minimal/none, 5−9 = mild, 10−14 = moderate, 15−21 = severe [34]. Scores ≥ 8 were categorized as probable anxiety. For this study, a cut‐off score of ≥ 8 was used to define “probable anxiety,” consistent with prior literature indicating this threshold optimally balances sensitivity and specificity in population‐level samples [29]. This classification is used to flag individuals likely to be experiencing clinically relevant anxiety symptoms. The GAD‐7 has been validated [35] and utilized in various populations [36], including agricultural workers in the Midwest [31, 37], showing good reliability and validity.

#### 2.6.5 Health

This section of the survey focused on assessing the health conditions of participants. To assess the prevalence of chronic health conditions, respondents were presented with the question: “Have you been diagnosed with any of the following health conditions? (Check all that apply.)” They were asked to check applicable conditions from a list of 11 common health conditions (listed in Table 5). To summarize overall health status, we created a binary variable identifying respondents as “healthy” if they reported no diagnosed conditions across any item, and “unhealthy” if they reported at least one diagnosis.

Participants were asked to evaluate their general health using a 5‐point scale, ranging from 0 (*poor*) to 4 (*excellent*). The question posed was: “How would you describe your general health? (Check the single most appropriate response).” Self‐rated health measures are commonly recognized as a reliable predictor of clinical outcomes and mortality [38].

#### 2.6.6 Agricultural Work‐Related Injury

Participants were asked, “Have you experienced an agricultural‐work injury in the past 6 months?” The response options were coded as follows: “Yes” (please continue to question 2), “No” (skip to the next section), and “Prefer not to disclose.” For those who responded “Yes,” further details about each incident were gathered.

Injury severity was measured using the item “How serious was the injury?” Participants were provided with response options: “Minor (requiring no medical treatment beyond bandage or antiseptic),” “Moderate (involving treatments like stitches or casts for broken bones, cuts, or sprains),” and “Severe (resulting in significant injuries such as amputation or other permanent disability).”

Injury location was assessed using the item “What part(s) of your body was injured? (Select all that apply)” with the following response options: “Arm,” “Eye,” “Genital,” “Leg,” “Finger,” “Hand,” “Neck,” “Toe,” “Trunk,” “Chest,” “Back,” “Foot,” “Head,” “Shoulder,” “Other” (specify), and “Prefer not to disclose.” Nature of injury was measured using the item “What was the nature of your most severe injury? (Select all applicable)” with the response options: “Amputation,” “Asphyxiation,” “Burn,” “Bruise,” “Fracture or broken bones,” “Cut/laceration,” “Eye injury,” “Puncture,” “Pinch,” “Sprain,” and “Other (specify).”

Participants also provided information on medical response and treatment received for their injuries through two multipart questions. The first question, “Who administered first aid?” allowed participants to select all applicable options from “Doctor,” “Family member,” “Nurse,” “Self‐treatment,” and “Other” (please specify). The second part asked, “Where were you treated?” with choices including “Clinic or outpatient department,” “Doctor's office,” “Home,” “Hospital, admitted (stayed at least one night),” “Hospital, emergency department,” and “No Treatment.” Following this, participants detailed the types of medical treatments received. They were asked, “What treatment was administered for the injury?” and could select all relevant responses from “Prescription medication,” “Surgery or an operation,” “Physical therapy or similar treatment,” ‘Stitches or cast,” ‘Not Applicable,” and “Other (please specify).”

#### 2.6.7 Data Analysis

Round 1 and Round 2 data were merged and analyzed using STATA v.18.0. Descriptive analyses were conducted to summarize participant demographics, agricultural injury prevalence, mental health measures, self‐reported general health, and chronic health conditions Results for categorical variables were reported as frequencies (*N*, %), while continuous variables were summarized using means (SD).

Associations between agricultural injury and stress, depression, anxiety, and chronic health conditions were evaluated using Pearson's chi‐squared test. Assumptions were examined by calculating expected cell counts. If any expected cell count was below five in 20% or more of the cells, Fisher's exact test was applied to ensure the validity of results. Statistical significance was set at *p* < 0.05.

## 3 Results

Across the two recruitment periods, 593 questionnaires were obtained from Illinois farmers, with 537 eligible respondents included in the final analysis.

### 3.1 Demographic and Farming Characteristics of Respondents

The mean age of participants was 61.4 years (SD = 13.4). The sample was predominantly male (89.2%), White (96.9%), and married (82.3%) (Table 1). About half (49.4%) identified as full‐time agricultural producers, while 35.2% reported primary income from off‐farm jobs. Field crops were the most reported primary commodity (81.9%) across the sample (Table 2).

**Table 1:** Demographic characteristics of respondents (*N* = 537).

Variable		*n* (%) or mean (SD)
Gender	Male	472 (89.2%)
	Female	57 (10.8%)
Age (years, mean ± SD)		61.4 (13.4)
Education	High school diploma	225 (41.9%)
	Technical or trade degree	47 (8.8%)
	Associate degree	90 (16.8%)
	Bachelor's degree	120 (22.4%)
	Master's degree or greater	46 (8.6%)
Race	White	532 (97.3%)
Marital status	Single	38 (7.1%)
	Married	444 (83.0%)
	Divorced/separated	26 (4.9%)
	Widowed/widower	27 (5.0%)

*Note:* Data combined across categories due to small cell size. The total number of respondents (*N*) varies by characteristic due to missing data.

**Table 2:** Farming characteristics of respondents.

Variable		*n* (%) or mean (SD)
Years farming as primary/secondary occupation		35.4 (16.3)
Employment type	Full‐time agricultural producer	266 (49.4%)
	Part‐time agricultural producer, primary income from farm/ranch	63 (11.7%)
	Part‐time agricultural producer, primary income from off‐farm job	189 (35.1%)
Farm type	Family or individual operation (excluding partnerships & corporations)	476 (87.2%)
	Partnership operation	33 (6.0%)
	Incorporated under the law	12 (2.2%)
	Other (such as trust, estate, etc.)	13 (2.4%)
Farm role	Principal or primary owner/operator (primary decision making)	361 (66.4%)
	Partner owner/operator (shared decision making)	125 (23.0%)
	Other	36 (6.6%)
Farm primary commodity	Field crops	474 (84.6%)
	Beef	52 (9.3%)
	Other	34 (6.1%)

*Note:* The total number of respondents (*N*) varies by characteristic due to missing data.

### 3.2 Perceived Stress and Mental Health

The mean PSS total score among respondents was 13.9 (SD = 6.4), with scores ranging from 0 to 36. The majority of participants (50.9%) reported low perceived stress (PSS scores 0–13), while 45.2% experienced moderate stress (scores 14–26), and 3.9% reported high perceived stress (scores 27–40) (Table 3).

**Table 3:** PSS category distribution (*N* = 537).

PSS stress category	Frequency (*n*)	Percent (%)
Low perceived stress (0−13)	273	50.8
Moderate stress (14−26)	243	45.3
High perceived stress (27−40)	21	3.9

Among PHQ‐9 respondents, 73.96% fell into the minimal depression category, 16.04% had mild depression, 6.04% experienced moderate depression, and 4.0% reported moderately severe or severe depression (Table 4). Using a validated screening threshold of PHQ‐9 ≥ 7 to indicate probable clinical depression, 18.30% (*n* = 97) of respondents screened positive. Among GAD‐7 respondents, 65.91% reported minimal anxiety, 22.79% had mild anxiety, 6.78% moderate anxiety, and 4.52% severe anxiety (Table 4). Using a cut‐off score of GAD‐7 ≥ 8 to indicate probable clinical anxiety, 16.95% (*n* = 90) screened positive (Table 4).

**Table 4:** PHQ‐9 and GAD‐7 category distribution.

		Frequency (*n*)	Percent (%)
PHQ‐9 category	Minimal depression (0−4)	392	73.96
	Mild depression (5−9)	85	16.04
	Moderate depression (10−14)	32	6.04
	Moderately severe depression (15−19)	14	2.64
	Severe depression (20−27)	7	1.32
PHQ‐9 screening classification ≥ 7	Below cut‐off	433	81.70
	At or above cut‐off	97	18.30
GAD‐7 category	Minimal anxiety (0−4)	350	65.91
	Mild anxiety (5−9)	121	22.79
	Moderate anxiety (10−14)	36	6.78
	Severe anxiety (15−21)	24	4.52
GAD‐7 screening classification ≥ 8	Below cut‐off	441	83.05
	At or above cut‐off	90	16.95

### 3.3 Physical Health

Most respondents reported good to very good general health. On a self‐reported health ranking scale, 44.8% rated their health as “good,” 35.2% as “very good,” and 9.4% as “excellent.” A smaller proportion reported fair (8.3%) or poor (1.3%) health, while 0.9% preferred not to disclose their health status.

Participants were asked whether they had been diagnosed with specific chronic health conditions (Table 5). Of the 530 valid responses, 227 participants (42.8%) reported no diagnosed health conditions, while 303 (57.2%) reported having at least one condition. The most commonly reported diagnoses were high cholesterol (27.4%), arthritis (21.3%), and hearing loss (23.4%). The least commonly reported condition was dementia or Alzheimer's disease (1.3%) (Table 6).

**Table 5:** Diagnosed health conditions and treatment among agricultural producers (*N* = 530).

Condition	Diagnosed *n* (%)	Of those diagnosed, how many receiving treatment *n* (%)	
		Receiving treatment *n* (%)	Not receiving treatment *n* (%)
Asthma	39 (7.4%)	14 (48.7%)	21 (53.8%)
Arthritis	113 (21.3%)	59 (52.2%)	52 (46.0%)
High blood pressure (hypertension)	84 (15.8%)	74 (88.1%)	7 (8.3%)
COPD	27 (5.1%)	15 (55.6%)	7 (25.9%)
High cholesterol	145 (27.4%)	127 (87.6%)	14 (9.7%)
Hearing loss	124 (23.4)	74 (59.7%)	43 (34.7%)
Heart condition	32 (6.1%)	29 (90.6%)	2 (6.3%)
Cancer	65 (12.3%)	33 (50.8%)	24 (36.9%)
Dementia/Alzheimer's disease	7 (1.3%)	2 (28.6%)	0 (0.0%)
Anxiety	45 (8.5%)	33 (73.3%)	7 (15.6%)
Depression	32 (6.0%)	21 (65.6%)	6 (18.8%)

*Note:* Diagnosed percentages are based on the total number of respondents (*N* = 530). Treatment percentages are based on the number of individuals who reported a diagnosis for each condition. Due to item nonresponse, some treatment data are missing, and therefore, totals may not sum perfectly.

**Table 6:** Characteristics of agricultural injuries among agricultural producers (*N* = 535).

Injury characteristic		*n*	Percent (%)
Injury in past 6 months	Yes	43	8.0
	No	492	91.6
Severity of agricultural injury	Minor	24	47.1
	Moderate	23	45.1
	Severe	1	2.0
Body part(s) injured in most severe injury	Arm	14	14.6
	Shoulder	10	10.4
	Leg	11	11.5
	Finger	15	15.6
	Hand	10	10.4
	Toe	3	3.1
	Back	12	12.5
	Foot	8	8.3
	Head	6	6.3
	Other	4	4.2
Nature of most severe injury	Amputation	1	1.5
	Burn	4	6.0
	Bruise	13	19.4
	Fracture or broken bones	6	9.0
	Cut/laceration	17	25.4
	Puncture	3	4.5
	Pinch	6	9.0
	Sprain	9	13.4
	Other	5	7.5
Medical provider for agricultural injury	Doctor	15	34.9
	Family member	5	11.6
	Nurse	2	4.7
	Self‐treatment	18	41.9
	Other	3	7.0
Treatment setting for agricultural injury	Clinic or outpatient department	6	15.0
	Doctor's office	8	20.0
	Home	14	35.0
	Hospital (admitted overnight)	3	7.5
	Hospital (emergency department)	5	12.5
	No treatment	4	10.0
Treatment type for agricultural injury	Prescription medication	11	25.0
	Surgery or operation	4	9.9
	Physical therapy or similar	8	18.2
	Stitches or cast	5	11.4
	Other	16	36.4

*Note:* Percentages are based on valid responses (*N* = 535). Participants could select multiple responses for body part(s), injury nature, and treatment. Totals may exceed 43 due to multiple selections by individual participants.

Among those who reported a diagnosed condition, treatment or medication use varied widely. Heart conditions, hypertension, and high cholesterol had the highest rates of treatment. In contrast, conditions such as asthma, arthritis, and cancer showed lower levels of treatment uptake, despite their relatively high prevalence.

### 3.4 Agricultural Work‐Related Injury

Among respondents, 8.0% (*n* = 43) reported experiencing an agricultural work injury in the past 6 months.

### 3.5 Health and Safety Associations

There were no significant associations between agricultural injury and stress levels (*p *= 0.125), depression severity (*p *= 0.885), or anxiety severity (*p *= 0.628) (Table 7). Although differences in stress and mental health conditions were observed, such as a higher proportion of individuals with injuries reporting mild depression (19.4% vs. 14.3%) and moderate anxiety (7.9% vs. 5.8%), these differences were not statistically significant.

**Table 7:** Association between agricultural injury, stress, and mental health.

Variable	No injury (*n*, %)	Injury (*n*, %)	*p* value
Stress category			0.125^a^
Low stress	227 (49.7%)	26 (66.7%)	
Moderate stress	212 (46.4%)	12 (30.8%)	
High stress	18 (3.9%)	1 (2.6%)	
PHQ‐9 depression severity			0.885^a^
Minimal depression	327 (76.4%)	27 (75.0%)	
Mild depression	61 (14.3%)	7 (19.4%)	
Moderate depression	26 (6.1%)	2 (5.6%)	
Moderate severe depression	9 (2.1%)	0 (0.0%)	
Severe depression	5 (1.2%)	0 (0.0%)	
PHQ‐9 screening classification ≥ 7			0.757
Below cut‐off	372 (82.7%)	33 (84.6%)	
At or above cut‐off	78 (17.3%)	6 (15.4%)	
GAD‐7 anxiety severity			
Minimal anxiety (0−4)	289 (67.4%)	27 (71.1%)	0.628^a^
Mild anxiety (5−9)	95 (22.1%)	8 (21.1%)	
Moderate anxiety (10−14)	25 (5.8%)	3 (7.9%)	
Severe anxiety (15−21)	20 (4.7%)	0 (0.0%)	
GAD‐7 screening classification ≥ 8			0.537
Below cut‐off	376 (83.4%)	34 (87.2%)	
At or above the cut‐off	75 (16.6%)	5 (12.8%)	

*Note:* Sample sizes vary due to missing data. Percentages reflect valid responses within each category. Fisher's exact test (^a^) used where expected cell counts were < 5.

Similarly, no significant associations were found between agricultural injury and chronic health conditions such as asthma (*p *= 0.171), arthritis (*p *= 0.557), high cholesterol (*p *= 0.413), hearing loss (*p *= 0.441), or cancer (*p *= 0.942) (Table 8). While individuals with agricultural injuries had higher observed rates of asthma (25.0% vs. 13.7%) and arthritis (45.8% vs. 39.7%), these differences were not statistically significant.

**Table 8:** Association between agricultural injury and chronic health conditions.

Health condition	No injury (*n*, %)	Injury (*n*, %)	*p* value
Asthma			0.171
Yes	29 (13.7%)	5 (25.0%)	
No	183 (86.3%)	15 (75.0%)	
Arthritis			0.557
Yes	96 (39.7%)	11 (45.8%)	
No	146 (60.3%)	13 (54.2%)	
COPD			0.228
Yes	24 (11.7%)	0 (0.0%)	
No	181 (88.3%)	16 (100%)	
High cholesterol			0.413
Yes	129 (50.0%)	9 (40.9%)	
No	129 (50.0%)	13 (59.1%)	
Hearing loss			0.441
Yes	110 (43.8%)	12 (52.2%)	
No	141 (56.2%)	11 (47.8%)	
Cancer			0.942
Yes	61 (27.1%)	5 (27.8%)	
No	165 (72.9%)	13 (72.2%)	
Dementia/Alzheimer's disease			1.000
Yes	7 (3.5%)	0 (0.0%)	
No	193 (96.0%)	16 (100%)	

## 4 Discussion

This study aimed to quantify the experience of stress, mental health symptoms, agricultural injuries, and the prevalence of chronic health conditions among Illinois farmers—a critical population in one of the nation's most agriculturally productive states. Our findings reveal several important insights and raise avenues for future inquiry.

### 4.1 Perceived Stress and Systemic Interventions

Approximately half of the respondents in our sample reported *moderate* or *high* perceived stress (mean = 13.9, SD = 6.4) on the PSS, a finding consistent with studies conducted among agricultural producers in the Western US and Canada [17, 25, 31, 39]. An examination of stress among agricultural producers in the Western US resulted in slightly higher mean PSS scores (M = 18.3, SD = 5.6), however, the sample was surveyed at a different time and was more diverse [25]. While individual perceived stress levels can fluctuate daily, the high proportion of the sample reporting at least moderate stress suggests that many farmers face chronic stressors with potential long‐term health consequences.

Given these findings, there is a continued need to implement interventions or policies aimed at reducing stress among farmers. Individual‐level programs—such as stress management workshops, counseling, and resilience training—can offer immediate support. However, many farmers identify financial pressures like high debt loads, escalating input costs, and volatile commodity prices as primary stressors, factors largely beyond individual control [17, 31, 40, 41, 42]. Thus, systemic interventions are also necessary. Policy initiatives and institutional programs that protect agricultural producers and stabilize financial conditions are essential to address the root causes of chronic stress in this population.

### 4.2 Mental Health Disparities and Barriers to Care

Approximately 18.30% of respondents met the criteria for probable depression (PHQ‐9 ≥ 7) and 16.95% for probable anxiety (GAD‐7 ≥ 8), rates that are comparable to or slightly lower than those observed in other agricultural populations, yet higher than general US estimates [17]. However, the proportion of respondents with a formal diagnosis was considerably lower (6.33% for depression and 8.75% for anxiety). The disparity between reported symptoms of anxiety and depression compared to diagnoses in our sample may be reflective of the barriers agricultural producers experience to accessing mental healthcare and help‐seeking behaviors among the population. System and institutional barriers include availability of mental health services in rural and agricultural communities, travel time to services, and costs associated with mental healthcare services [43, 44]. Social and cultural barriers include stigma associated with seeking care for mental health and emphasis on personal characteristics such as stoicism and self‐reliance in the agricultural community [45].

### 4.3 Agricultural Injuries: Prevalence, Costs, and Research Needs

About 8% (*n* = 43) reported experiencing an agricultural work‐related injury in the past 6 months. Nearly half of these injuries were moderate or severe. Injuries affected various body parts relatively equally, with cuts, lacerations, or bruises being the most common. Less than 10% of injuries required surgery or an operation, and 7.5% resulted in hospitalization. These injury characteristics are similar to those reported in other Midwest populations. For example, Johnson et al. reported that 5.8% of farmers and ranchers in Iowa, Minnesota, North Dakota, South, Dakota, Nebraska, and Kansas experienced a work‐related injury in the previous year, with 56% requiring a doctor visit and 12% requiring hospitalization [46].

In 2019, an agricultural injury was estimated to cost $10,878 in medical care, and $4735 in lost work time, for a total of $15,613 [47]. While our survey did not capture detailed contextual information surrounding these injuries, the similarities in injury profiles observed in our sample and that of Adhikari et al. [47] suggest that the associated costs may be comparable. These potential medical costs and lost work time can significantly affect the financial well‐being and viability of a farm, particularly during economic downturns. Future research should directly estimate the costs of agricultural injuries in Illinois and communicate these findings to farmers.

Unfortunately, our results offer limited insight into the circumstances and precipitating events of these injuries. Such contextual details are challenging to capture via a survey alone and may be better addressed through qualitative methods, where follow‐up questions can explore the events leading to injury more deeply. A mixed methods approach is recommended to quantify injury characteristics but also gather in‐depth narratives about precipitating events and conditions [48].

### 4.4 Relationship Between Stress and Injury

Among agricultural producers in our sample, perceived stress level was not associated with agricultural injury. This finding contrasts with previous research suggesting that higher stress levels increase the risk of farm‐related injuries [49]. Similarly, Bai et al. found high perceived stress level as a risk factor for injury [14]. Most studies examining the relationship between stress and injury are cross‐sectional, and the temporal relationship cannot be ascertained. Stress may contribute to work‐related injuries by impairing concentration, decision making, and physical coordination [50]. There is evidence to suggest that individuals that are stressed may be less attentive to safety protocols and engage in risky behaviors [50, 51]. Given the potential consequences of injuries—including loss of life, lost work time, and reduced productivity—it remains critical for agricultural producers to prioritize effective stress reduction and management strategies.

### 4.5 Chronic Health Conditions & Treatment Among Agricultural Producers

While prior research has examined work‐related injuries among farmers [52], chronic health conditions have received less attention. In our sample, 27.4% of respondents reported a diagnosis of high cholesterol, 23.4% reported hearing loss, and 15.8% reported high blood pressure. Additionally, 21.3% reported arthritis, 7.4% reported asthma, 5.1% reported chronic obstructive pulmonary disease (COPD), 6.1% reported a heart condition, and 12.3% reported cancer.

Although the data on these conditions are somewhat dated, previous research indicates that agricultural producers experience conditions such as cardiovascular disease, arthritis, and hearing loss at rates similar to or higher than those of other employed populations [53]. Yet, findings from the Agricultural Health Study reveal that, compared to the general population, farmers have a reduced risk of mortality from cardiovascular diseases, diabetes, and total cancer [54]. These results may be described by the healthy worker effect and contribute to anecdotal observations that farmers and ranchers are a healthy subpopulation in the United States. Importantly, the results from this study present farmers with conditions, not having died from the condition.

Regarding treatment uptake, the proportion of farmers receiving care for their chronic conditions varied considerably. For instance, a high percentage of those diagnosed with high blood pressure (88.1%), high cholesterol (87.6%), and heart conditions (90.6%) reported receiving treatment. In contrast, treatment rates for asthma (48.7%), cancer (50.8%), and arthritis (52.2%) were notably lower, indicating that a substantial segment of farmers with these conditions remains untreated. These discrepancies likely reflect differences in healthcare access, provider availability, and cultural factors that influence help‐seeking behaviors in rural settings. Future research should explore these barriers in depth and develop targeted interventions to improve treatment uptake, thereby enhancing the overall health and well‐being of agricultural producers.

### 4.6 Limitations

This study provides information around the types of injuries and chronic health conditions experienced by farmers in Illinois, providing guidance to extension, healthcare, and public health for programming and services. However, several limitations should be noted.

The mailed survey yielded response rates comparable to other studies of Midwestern agricultural producers [46]. However, almost 90% of respondents reported field crops as their primary commodity, and 6% reported beef cattle. While these figures align with the 2022 Census of Agriculture [55], the results do not capture the full spectrum of agricultural production or associated hazardous exposures. Moreover, this study largely represents the injury experience of males in Illinois. These results are not generalizable to farmers and ranchers in other geographies or production systems that encounter different exposures and therefore, a different injury profile. These results are not generalizable to women or farmworkers. Future research should consider the injury experience of farm women and farm labor.

The cross‐sectional design of the study further limits our ability to draw causal inferences between variables. Future research should incorporate longitudinal approaches to better understand the temporal relationships between occupational exposures and health outcomes. Additionally, as with many observational studies, our results may be influenced by recall and selection biases. A strength of this study was the use of validated instruments to assess stress (PSS) and screen for anxiety (GAD‐7) and depression PHQ‐9), the prescribed recall periods for each—ranging from 2‐weeks to 6‐months—may have resulted in a null finding when a significant relationship between stress, mental health, and health existed. Future research should consider standardizing instruments used to assess or measure stress and mental health symptomology to meaningful comparisons can be made across populations or over time.

Despite these limitations, this study provides a contemporary examination of the health and safety challenges faced by agricultural producers in Illinois and underscores the need for further research to expand our understanding of these critical issues.

## Author Contributions

Josie Rudolphi, Salah Issa, and Courtney Cuthbertson conceptualized the work and received funding to support the project. Josie Rudolphi, Courtney Cuthbertson, and Salah Issa developed the survey instrument and collected data. Kaleigh Barnett directed data entry and data analysis. Josie Rudolphi and Kaleigh Barnett drafted the manuscript. Courtney Cuthbertson and Salah Issa edited and revised the manuscript. All authors agree to be accountable for all aspects of the work in ensuring that questions related to the accuracy or integrity of any part of the work are appropriately investigated and resolved.

## Disclosure

The authors have nothing to report.

## Ethics Statement

This study was approved by the University of Illinois Urbana‐Champaign Institutional Review Board.

## Consent

Written informed consent was obtained from all participants.

## Conflicts of Interest

The authors declare no conflicts of interest.

## References

[1] C. Dimitri , A. Effland , and N. Conklin , “The 20th Century Transformation of US Agriculture and Farm Policy,” 2005.

[2] M. Aso , “226 Worker Health in Modern Agriculture,” in The Oxford Handbook of Agricultural History, ed. J. Whayne (Oxford University Press, 2024).

[3] J. R. Dimple , Indu , and M. Kumar , “Human Health Hazards and Risks in the Agriculture Sector,” in Recent Technologies for Disaster Management and Risk Reduction: Sustainable Community Resilience & Responses, eds. P. K. Rai , P. Singh , and V. N. Mishra (Springer International Publishing, 2021), 229–244.

[4] J. E. Lessenger and S. H. Schuman , Agricultural Medicine: A Practical Guide (Springer, 2006).

[5] B. E. Todd and R. M. Buchan , “Total Dust, Respirable Dust, and Microflora Toxin Concentrations in Colorado Corn Storage Facilities,” Applied Occupational and Environmental Hygiene 17, no. 6 (2002): 411–415. 12049430 10.1080/10473220290035426

[6] T. H. Y. Nguyen , M. Bertin , J. Bodin , N. Fouquet , N. Bonvallot , and Y. Roquelaure , “Multiple Exposures and Coexposures to Occupational Hazards Among Agricultural Workers: A Systematic Review of Observational Studies,” Safety and Health at Work 9, no. 3 (2018): 239–248. 30370155 10.1016/j.shaw.2018.04.002 PMC6129995

[7] S. Li , M. M. S. Raza , and S. Issa , “Agricultural Injury Surveillance in the United States and Canada: A Systematic Literature Review,” Journal of Agromedicine 29, no. 2 (2024): 122–135. 38251421 10.1080/1059924X.2024.2304699

[8] Bureau of Labor Statistics , “Employer‐Reported Workplace Injuries and Illnesses,” 2023,

[9] US Bureau of Labor Statistics , “Nonfatal Occupational Injuries and Illnesses. Injuries, Illnesses, and Fatalities,” 2021, https://www.bls.gov/iif/soii-overview.htm.

[10] K. Clarke , A. Manrique , T. Sabo‐Attwood , and E. S. Coker , “A Narrative Review of Occupational Air Pollution and Respiratory Health in Farmworkers,” International Journal of Environmental Research and Public Health 18, no. 8 (2021): 4097. 33924663 10.3390/ijerph18084097 PMC8070429

[11] J. Y. Kang , S. W. Song , H. Hong , et al., “Clinical Characteristics and Outcomes of Injuries in Agricultural and Nonagricultural Workers Visiting the Emergency Department: A Propensity‐Matched Analysis,” Clinical and Experimental Emergency Medicine 11, no. 1 (2024): 68–78. 37439139 10.15441/ceem.23.022 PMC11009704

[12] J. Runkle , J. Flocks , J. Economos , J. Tovar‐Aguilar , and L. McCauley , “Occupational Risks and Pregnancy and Infant Health Outcomes in Florida Farmworkers,” International Journal of Environmental Research and Public Health 11, no. 8 (2014): 7820–7840. 25101767 10.3390/ijerph110807820 PMC4143835

[13] B. Salami , S. Meharali , and A. Salami , “The Health of Temporary Foreign Workers in Canada: A Scoping Review,” Canadian Journal of Public Health 106, no. 8 (2015): e546–e554. 10.17269/CJPH.106.5182 PMC6972066 26986918

[14] H. Bai , C. Beseler , L. Baccaglini , and R. H. Rautiainen , “Work‐Related Stress as a Risk Factor for Farm Injuries in the Central United States,” Journal of Agricultural Safety and Health 29, no. 1 (2023): 1–13. 40079952 10.13031/jash.14951

[15] E. M. Furey , D. O'Hora , J. McNamara , S. Kinsella , and C. Noone , “The Roles of Financial Threat, Social Support, Work Stress, and Mental Distress in Dairy Farmers' Expectations of Injury,” Frontiers in Public Health 4 (2016): 126. 27446893 10.3389/fpubh.2016.00126 PMC4914495

[16] C. L. Beseler and L. Stallones , “Safety Knowledge, Safety Behaviors, Depression, and Injuries in Colorado Farm Residents,” American Journal of Industrial Medicine 53, no. 1 (2010): 47–54. 19921707 10.1002/ajim.20779

[17] J. M. Rudolphi , C. Cuthbertson , A. Kaur , and J. Sarol , “A Comparison Between Farm‐Related Stress, Mental Health, and Social Support Between Men and Women Farmers,” International Journal of Environmental Research and Public Health 21, no. 6 (2024): 684. 38928931 10.3390/ijerph21060684 PMC11204078

[18] Farm Market iD , “Objective Insights on Both Growers & Farms,” 2020, https://www.farmmarketid.com/data/.

[19] D. A. Dillman , J. D. Smyth , and L. M. Christian , Internet, Phone, Mail, and Mixed‐Mode Surveys: The Tailored Design Method, 4th ed. (Wiley, 2014).

[20] S. Cohen , T. Kamarck , and R. Mermelstein , “A Global Measure of Perceived Stress,” Journal of Health and Social Behavior 24, no. 4 (1983): 385–396. 6668417

[21] S. Cohen and G. M. Williamson , “Perceived Stress in a Probability Sample of the United States.” The Social Psychology Of Health (Sage, 1988).

[22] Q. Hu , M. Tuluhong , and P. Han , “Odor Awareness Modulates the Association Between Perceived Stress and Chemosensory Anhedonia in Women,” PsyCh Journal 13, no. 5 (2024): 870–879. 38757253 10.1002/pchj.769 PMC11444723

[23] P. García‐Martínez , R. Ballester‐Arnal , K. Gandhi‐Morar , et al., “Perceived Stress in Relation to Quality of Life and Resilience in Patients With Advanced Chronic Kidney Disease Undergoing Hemodialysis,” International Journal of Environmental Research and Public Health 18, no. 2 (2021): 536. 33440671 10.3390/ijerph18020536 PMC7826655

[24] D. A. Pizzagalli , R. Bogdan , K. G. Ratner , and A. L. Jahn , “Increased Perceived Stress Is Associated With Blunted Hedonic Capacity: Potential Implications for Depression Research,” Behaviour Research and Therapy 45, no. 11 (2007): 2742–2753. 17854766 10.1016/j.brat.2007.07.013 PMC2080833

[25] M. Grocke‐Dewey , A. Brennan , B. Freeman , et al., Perceived Stress, Stressors, and Preferred Stress Management Strategies Among Western Agricultural Producers (Educational Publishing Foundation, 2023), 10.1037/rmh0000233.

[26] B. Löwe , K. Kroenke , W. Herzog , and K. Gräfe , “Measuring Depression Outcome With a Brief Self‐Report Instrument: Sensitivity to Change of the Patient Health Questionnaire (PHQ‐9),” Journal of Affective Disorders 81, no. 1 (2004): 61–66. 15183601 10.1016/S0165-0327(03)00198-8

[27] M. P. Pignone , B. N. Gaynes , J. L. Rushton , et al., “Screening for Depression in Adults: A Summary of the Evidence for the US Preventive Services Task Force,” Annals of Internal Medicine 136, no. 10 (2002): 765–776. 12020146 10.7326/0003-4819-136-10-200205210-00013

[28] K. Kroenke , R. L. Spitzer , and J. B. W. Williams , “The PHQ‐9: Validity of a Brief Depression Severity Measure,” Journal of General Internal Medicine 16, no. 9 (2001): 606–613. 11556941 10.1046/j.1525-1497.2001.016009606.x PMC1495268

[29] D. Villarreal‐Zegarra , J. Barrera‐Begazo , S. Otazú‐Alfaro , N. Mayo‐Puchoc , J. C. Bazo‐Alvarez , and J. Huarcaya‐Victoria , “Sensitivity and Specificity of the Patient Health Questionnaire (PHQ‐9, PHQ‐8, PHQ‐2) and General Anxiety Disorder Scale (GAD‐7, GAD‐2) for Depression and Anxiety Diagnosis: A Cross‐Sectional Study in a Peruvian Hospital Population,” BMJ Open 13, no. 9 (2023): e076193. 10.1136/bmjopen-2023-076193 PMC10510859 37714674

[30] J. Arrieta , M. Aguerrebere , G. Raviola , et al., “Validity and Utility of the Patient Health Questionnaire (PHQ)‐2 and PHQ‐9 for Screening and Diagnosis of Depression in Rural Chiapas, Mexico: A Cross‐Sectional Study: PHQ‐9 Validity for Depression Diagnosis,” Journal of Clinical Psychology 73, no. 9 (2017): 1076–1090. 28195649 10.1002/jclp.22390 PMC5573982

[31] J. M. Rudolphi , R. L. Berg , and A. Parsaik , “Depression, Anxiety and Stress Among Young Farmers and Ranchers: A Pilot Study,” Community Mental Health Journal 56, no. 1 (2020): 126–134. 31583619 10.1007/s10597-019-00480-y

[32] A. Bjornestad , L. Brown , and L. Weidauer , “The Relationship Between Social Support and Depressive Symptoms in Midwestern Farmers,” Journal of Rural Mental Health 43, no. 4 (2019): 109–117.

[33] B. Levis , Y. Sun , C. He , et al., “Accuracy of the PHQ‐2 Alone and in Combination With the PHQ‐9 for Screening to Detect Major Depression: Systematic Review and Meta‐Analysis,” Journal of the American Medical Association 323, no. 22 (2020): 2290–2300. 32515813 10.1001/jama.2020.6504 PMC7284301

[34] R. L. Spitzer , K. Kroenke , J. B. W. Williams , and B. Löwe , “A Brief Measure for Assessing Generalized Anxiety Disorder: The GAD‐7,” Archives of Internal Medicine 166, no. 10 (2006): 1092–1097. 16717171 10.1001/archinte.166.10.1092

[35] S. Kertz , J. Bigda‐Peyton , and T. Bjorgvinsson , “Validity of the Generalized Anxiety Disorder‐7 Scale in an Acute Psychiatric Sample,” Clinical Psychology & Psychotherapy 20, no. 5 (2013): 456–464. 22593009 10.1002/cpp.1802

[36] S. U. Johnson , P. G. Ulvenes , T. Øktedalen , and A. Hoffart , “Psychometric Properties of the General Anxiety Disorder 7‐Item (GAD‐7) Scale in a Heterogeneous Psychiatric Sample,” Frontiers in Psychology 10 (2019): 1713. 31447721 10.3389/fpsyg.2019.01713 PMC6691128

[37] A. Bjornestad , C. Cuthbertson , and J. Hendricks , “An Analysis of Suicide Risk Factors Among Farmers in the Midwestern United States,” International Journal of Environmental Research and Public Health 18, no. 7 (2021): 3563. 33808131 10.3390/ijerph18073563 PMC8036405

[38] M. Jylhä , “What Is Self‐Rated Health and Why Does It Predict Mortality? Towards a Unified Conceptual Model,” Social science & medicine (1982) 69, no. 3 (2009): 307–316. 19520474 10.1016/j.socscimed.2009.05.013

[39] A. Jones‐Bitton , C. Best , J. MacTavish , S. Fleming , and S. Hoy , “Stress, Anxiety, Depression, and Resilience in Canadian Farmers,” Social Psychiatry and Psychiatric Epidemiology 55, no. 2 (2019): 229–236. 31197397 10.1007/s00127-019-01738-2

[40] R. S. Paxton , Stress in Farming Communities: Making Best Use of Existing Help. North Tyneside & Northumberland NHS Trust, 2000).

[41] K. M. Gunn , L. J. Kettler , G. L. Skaczkowski , and D. A. Turnbull , “Farmers' Stress and Coping in a Time of Drought,” Rural and Remote Health 12 (2071): 2012. 23067269

[42] M. A. Mitten and P. Molenberghs , “Stress and Coping in Australian Male Farmers,” Australian Journal of Rural Health 33, no. 1 (2025): e13207. 39686619 10.1111/ajr.13207

[43] G. Murray , F. Judd , H. Jackson , et al., “Rurality and Mental Health: The Role of Accessibility,” Australian & New Zealand Journal of Psychiatry 38, no. 8 (2004): 629–634. 15298585 10.1080/j.1440-1614.2004.01426.x

[44] B. Brew , K. Inder , J. Allen , M. Thomas , and B. Kelly , “The Health and Wellbeing of Australian Farmers: A Longitudinal Cohort Study,” BMC Public Health 16 (2016): 988. 27634298 10.1186/s12889-016-3664-y PMC5025556

[45] M. J. Hull , K. M. Gunn , A. E. Smith , M. Jones , and J. Dollman , “‘We'Re Lucky to Have Doctors at All’; A Qualitative Exploration of Australian Farmers' Barriers and Facilitators to Health‐Related Help‐Seeking,” International Journal of Environmental Research and Public Health 19, no. 17 (2022): 11075. 36078793 10.3390/ijerph191711075 PMC9517750

[46] A. Johnson , L. Baccaglini , G. R. Haynatzki , C. Achutan , D. Loomis , and R. H. Rautiainen , “Agricultural Injuries Among Farmers and Ranchers in the Central United States During 2011‐2015,” Journal of Agromedicine 26, no. 1 (2021): 62–72. 33131463 10.1080/1059924X.2020.1845268

[47] S. Adhikari , F. Wilson , and R. Rautiainen , “Cost of Agricultural Injuries in the United States: Estimates Based on Surveillance, Insurance, and Government Statistics,” American Journal of Industrial Medicine 67, no. 9 (2024): 801–812. 38922747 10.1002/ajim.23628

[48] T. Stickley , A. O'Caithain , and C. Homer , “The Value of Qualitative Methods to Public Health Research, Policy and Practice,” Perspectives in Public Health 142, no. 4 (2022): 237–240. 35362352 10.1177/17579139221083814 PMC9446427

[49] K. Simpson , R. Sebastian , T. E. Arbuckle , C. Bancej , and W. Pickett , “Stress on the Farm and Its Association With Injury,” Journal of Agricultural Safety and Health 10, no. 3 (2004): 141–153. 15461131 10.13031/2013.16471

[50] A. J. Porcelli and M. R. Delgado , “Stress and Decision Making: Effects on Valuation, Learning, and Risk‐Taking,” Current Opinion in Behavioral Sciences 14 (2017): 33–39. 28044144 10.1016/j.cobeha.2016.11.015 PMC5201132

[51] Z. Yuan , Y. Li , and J. Lin , “Linking Challenge and Hindrance Stress to Safety Performance: The Moderating Effect of Core Self‐Evaluation,” Personality and Individual Differences 68 (2014): 154–159.

[52] D. I. Douphrate , L. Stallones , C. Lunner Kolstrup , et al., “Work‐Related Injuries and Fatalities on Dairy Farm Operations: A Global Perspective,” Journal of Agromedicine 18, no. 3 (2013): 256–264. 23844792 10.1080/1059924X.2013.796904

[53] R. M. Brackbill , L. L. Cameron , and V. Behrens , “Prevalence of Chronic Diseases and Impairments Among US Farmers, 1986‐1990,” American Journal of Epidemiology 139, no. 11 (1994): 1055–1065. 8192138 10.1093/oxfordjournals.aje.a116949

[54] A. Blair , D. P. Sandler , R. Tarone , et al., “Mortality Among Participants in the Agricultural Health Study,” Annals of Epidemiology 15, no. 4 (2005): 279–285. 15780775 10.1016/j.annepidem.2004.08.008

[55] United States Department of Agriculture and National Agricultural Statistics Service , “2022 Census of Agriculture,” 2024.

